# Understanding the mechanisms underlying phase-locking behavior in the crayfish swimmeret system

**DOI:** 10.1186/1471-2202-12-S1-O14

**Published:** 2011-07-18

**Authors:** Timothy J Lewis, Jiawei Zhang, Carmen Smarandache, Brian Mulloney

**Affiliations:** 1 Department of Mathematics, University of California, Davis, CA 95616, USA; 2 Department of Neurobiology, Physiology and Behavior, University of California, Davis, CA, 95616, UK

## 

One of the fundamental challenges in neuroscience is to understand how the intrinsic properties of neurons and the properties of neural networks combine to produce behavior. Networks that produce rhythmic motor behaviors, such as locomotion, provide important model systems to address this problem. A particularly good model for this purpose is the neural circuit underlying the coordinated rhythmic limb movements in the crayfish swimmeret system.

During forward swimming, rhythmic movements of swimmerets on different segments of the crayfish abdomen progress from back to front with the same period, but neighboring swimmerets are phase-lagged by 25% of the period. This coordination of limb movements is maintained over a wide range of frequency. The exact mechanisms underlying this robustly stable phase-locking are not known. Here, we use mathematical modeling and analysis in conjunction with recent experimental results to obtained insight into these mechanisms.

The rhythmic behavior of each swimmeret is driven by a local pattern generating circuit consisting of a half-center oscillator (HCO). These local pattern generating circuits are connected through well-described intersegmental connections. We model the neural circuitry of the swimmeret system as a chain of HCOs. First, we examine the phase response properties of HCOs for two fundamentally different mechanisms that produce anti-phase activity in HCOs: the “escape” and “release” mechanisms. We demonstrate that the “escape” and “release” mechanisms give rise to very different phase response properties, and we use phase plane arguments to explain the different shapes of the phase response curves. We then examine a chain of four nearest-neighbor coupled HCOs. We use the coupled oscillator theory and symmetry arguments to show that the phase-locking 25% phase-locking observed in the crayfish swimmeret system arises naturally from the network connectivity, but this phase-locking is robustly stable for only some combinations of connectivity and the escape/release mechanisms.

